# The frequency and persistence of lymphedema diagnosis and self-reported symptoms over 5 years in patients with endometrial carcinoma

**DOI:** 10.1016/j.gore.2022.100996

**Published:** 2022-05-07

**Authors:** Julia Ritchie, Quetrell Heyward, Nicholas Taylor, Emily Ko, Ashley F. Haggerty, Ashley Graul

**Affiliations:** a Division of Gynecologic Oncology, University of Pennsylvania Health System, Philadelphia, PA, USA; b Department of Obstetrics and Gynecology, University of Pennsylvania Health System, Philadelphia, PA, USA; c Division of Gynecologic Oncology, St. Luke’s University Health Network, Bethlehem, PA, USA

**Keywords:** Lymphedema, Endometrial cancer, Persistent symptoms

## Abstract

A potentially debilitating sequela of diagnosis or treatment for endometrial cancer islower limb lymphedema (LLL), which can have significant impacts on quality of life. Theobjective of this study was to determine the frequency of LLL symptoms in uterinecancer survivors over a 5-year study period. An IRB-approved prospective study of quality of life of endometrial cancer patients whounderwent surgical intervention was undertaken. The Gynecologic CancerLymphedema Questionnaire (GCLQ) was used to survey patients in 2011 and again in2016 to evaluate for symptoms of LLL.205 patients initially answered the survey, and 75 patients completed the follow upsurvey as well, with no differences in demographics between the cohorts. 90.7% ofpatients underwent lymph node dissection. Patients commonly reported symptoms ofnumbness (66.83%), aching (54.2%), and poor physical function (47.8%). On initialsurvey, 14.7% (n = 11) of patients met criteria for LLL by GCLQ criteria, with 8 patientsreporting improvement in symptoms and 3 reporting persistent diagnosis at follow up. At follow up survey, 12.0% (n = 9) patients meeting criteria five years later, with 6patients newly meeting criteria. The most persistent symptoms were poor physicalfunction (70.6%), numbness (72.5%), general swelling (55.6%), aching (64.1%), andlimb-related swelling (60%).While the rate of LLL was similar to previous reports, there were a number of newdiagnoses of LLL at interval follow up distant from surgery, up to 7 years later.Symptoms of LLL also persisted for many years after diagnosis.

## Highlights

• Numbness, aching, and poor physical function were commonly reported symptoms in endometrial cancer patients.
• Lymphedema symptoms that develop soon after diagnosis of endometrial cancer can persist for at least 5 years.
• Lymphedema symptoms could develop up to 7 years after initial diagnosis or treatment for endometrial cancer.

## 1 Introduction

Approximately 3.1% of women will be diagnosed with endometrial cancer at some point during their lifetime, with 813,861 women living with endometrial cancer in the United States in 2018. (xxxx) Despite early diagnosis and favorable 5-year survival rates (81.1%) (xxxx), many uterine cancer survivors develop detrimental sequelae associated with cancer treatment such as surgery, lymphadenectomy, chemotherapy, and adjuvant pelvic radiation.

One of the notable and debilitating sequelae of diagnosis or treatment for endometrial cancer is secondary lower limb lymphedema (LLL). LLL occurs when there is a disruption of lymphatic transport that can be either considered primary or secondary. Primary LLL is usually congenitally acquired while secondary LLL occurs due to anatomical disruption. (Dessources et al., 2020) Among secondary causes of LLL, operative dissection and radiation are common and reported to result in LLL in 5%-34.5% of cancer survivors following gynecological cancer treatment. (Abu-Rustum et al., 2006, Yost et al., 2014, Beesley et al., 2007, Brown et al., 2013, Ryan et al., 2003) A high proportion of patients in the literature that had LLL were noted to be endometrial cancer survivors (8–23%). (Abu-Rustum et al., 2006, Yost et al., 2014, Beesley et al., 2007) The diagnosis of LLL is important as it can adversely impact patient’s lives with significant financial burdens, alterations in daily activities or functions of daily living, an increased risk for depression, anxiety, and create a negative body image. (Ryan et al., 2003) Many women need to purchase new equipment, or change their wardrobe or activities to cope with their new diagnosis of LLL. (Ryan et al., 2003) As was shown in the GOG 244 LeG study, these symptoms also correlated with more cancer distress and worse sexual and vaginal health. (Carter et al., 2021).

There is no current gold standard method to diagnose LLL; however, literature has explored circumferential measuring of the lower limbs, water volumetry, lymphoscintigraphy and other imaging studies, as well as simple physical exam and history to aid in diagnosis. (Dessources et al., 2020) The Gynecologic Cancer Lymphedema Questionnaire (GCLQ) has been adopted for routine clinical care and validated against circumferential measures of the lower limbs, and has a predictive value in early onset LLL or those at risk of developing the condition. (Carter et al., 2021) Patient reported outcomes (PRO) by GCLQ define a greater symptom burden of LLL, and are more informative than circumferential measurements in demonstrating the impact on the patient. (Carter et al., 2021).

Early treatment of LLL is essential in preventing progression while late stages of LLL may cause severe physical, (Brown et al., 2013, Franks et al., 2006) and psychological problems, (Jager et al., 2006) as well as a deterioration in a patient’s quality of life. (Kim et al., 2015, Finnane et al., 2011) To date, no study has looked at the longitudinal symptoms of LLL among gynecologic cancer patients. We therefore sought to evaluate LLL symptoms in a longitudinal cohort of endometrial cancer survivors.

## 2 Methods

We conducted an IRB-approved prospective survey of endometrial cancer patients at the Hospital of the University of Pennsylvania in Philadelphia, Pennsylvania. Participants included women > 20 years old with a history of endometrial cancer who were undergoing surgical intervention. Participants were identified using gynecologic oncology fellow surgical case logs from 2008 to 2010 and ICD-9 diagnosis codes 179.0 and 182.0–182.8 from 2006 to 2010.

Participants were initially surveyed in 2011 regarding multiple aspects of survivorship and quality of life. A letter was sent to eligible participants by their oncologist explaining the purpose of the study. Informed consent was solicited for participation in the study and was obtained from each participant, utilizing consent form approved by IRB. The 205 women who responded to the initial survey in 2011 were re-surveyed in a similar fashion in 2016. This study was restricted to subjects’ answers to the GCLQ portion of the survey.

The GCLQ is utilized to assess symptoms associated with LLL. The GCLQ is a validated, self-report measure that assesses seven domains of symptoms in the lower extremities. These include heaviness, general swelling, limb-related swelling, infection, aching, numbness, and physical function. Participants reporting ≥ 5 symptoms of the lower extremities within the 7 listed domains were classified as having LLL. (Carter et al., 2021).

Descriptive statistics were performed on baseline demographics and GCLQ survey domains. Fisher's exact test and Wilcoxan rank sum tests were used to evaluate the potential differences. Hypothesis testing was conducted using a two-sided significance level of α = 0.05. Normality of continuous variables was determined and appropriate analysis was completed to determine difference in survey domains in the initial survey compared to follow up survey. Further analysis using chi-squared test compared the initial patient reported diagnosis of LLL to follow up diagnosis of LLL as well as Mcnemar tests to determine change in proportion in paired data.

## 3 Results

Demographic and clinical characteristics of study participants are depicted in Table 1. Of the original 205 patients that answered the initial survey, 75 completed both the initial and follow-up survey (36.6% response rate). The time from diagnosis to initial survey was 2.2 ± 1.2 years with a follow up survey completed at 7.2 ± 1.2 years after initial. There were no noted differences in demographics between the entire cohort and follow up respondents. The mean age of respondents at diagnosis of endometrial cancer of this cohort was 61.5 years with a mean BMI of 28.3 kg/m^2^. The most common histologic diagnosis and stage was endometrioid (72.0%) and stage 1A (60.0%). The majority of patients underwent lymph node dissection (90.7%) with an average of 16.0 lymph nodes (IQR 7–24) collected from each patient. A minority of patients received subsequent adjuvant radiation (48.0%) and chemotherapy (26.7%).

**Table 1:** Baseline demographics.

	**All Patients n = 205n (%)**	**Completed Initial and Follow-up Survey n = 75n (%)**	**p-value**
**Age at diagnosis**	61.0 (55.0, 66.0)	61.5 (57.0, 67.0)	0.48
**BMI at diagnosis**	30.5 (24.4, 36.8)	28.3 (23.5, 36.6)	0.19
**Race**			
White	167 (81.5%)	63 (84.0%)	0.36
Black	21 (10.2%)	4 (5.3%)	
Asian	8 (3.9%)	5 (6.7%)	
other	4 (2.0%)	0 (0.0%)	
unknown	5 (2.4%)	3 (4.0%)	
**Menopause**			
No	38 (18.5%)	13 (17.3%)	0.82
Yes	167 (81.5%)	62 (82.7%)	
**Histology**			
Endometrioid	150 (73.2%)	54 (72.0%)	0.96
Non-endometrioid	46 (22.4%)	18 (24.0%)	
Mixed	9 (4.4%)	3 (4.0%)	
**Stage**			
1A	132 (64.4%)	45 (60.0%)	0.84
1B	31 (15.1%)	16 (21.3%)	
2	12 (5.9%)	5 (6.7%)	
3A	17 (8.3%)	6 (8.0%)	
3C	8 (3.9%)	2 (2.7%)	
4B	5 (2.4%)	1 (1.3%)	
**Lymph Node Dissection**			
No	26 (12.7%)	7 (9.3%)	0.44
Yes	179 (87.3%)	68 (90.7%)	
**#Lymph Nodes**	12.0 (6.0, 19.0)	16.0 (7.0, 24.0)	0.17
**Time from diagnosis to survey**	2.2 (1.6, 3.8)	2.2 (1.6, 3.6)	0.97
**Comorbidities**			
Hypertension	90 (43.9%)	30 (40.0%)	
Diabetes	31 (15.1%)	9 (12.0%)	
Cardiac Disease	55 (26.8%)	18 (24.0%)	
Renal Disease	7 (3.4%)	3 (4.0%)	
Liver Disease	6 (2.9%)	3 (4.0%)	
**Chemotherapy**			
None	140 (68.3%)	53 (70.7%)	0.64
Yes	57 (27.8%)	20 (26.7%)	
Yes, at recurrence	4 (2.0%)	0 (0.0%)	
Yes, other cancer	4 (2.0%)	2 (2.7%)	
**Radiation**			
None	123 (60.0%)	39 (52.0%)	0.41
Yes	79 (38.5%)	36 (48.0%)	
Yes, at recurrence	1 (0.5%)	0 (0.0%)	
Yes, other cancer	2 (1.0%)	0 (0.0%)	

GCLQ results among subjects are noted in Table 2. The most common symptoms reported in the initial GCLQ survey among the entire cohort were numbness (66.8%), aching (54.2%), and poor physical function (47.8%). There were no significant differences among the initial survey responses of those who completed only the initial survey versus those who completed both surveys. For those that also completed the follow-up survey 5 years later, numbness (60.0%), aching (52.0%), and poor physical function (58.7%) continued to be the most commonly reported symptoms. Although not statistically significant, the number of people reporting numbness decreased over the 5 years from 68% to 60%. A similar trend can be seen with reported swelling (36% to 30.7%). On the other hand, the number of people who reported a decline in physical function showed an increase from 45.3% to 58.7 %By GCLQ criteria, 14.7% of patients who answered both surveys met the diagnosis of LLL (11 of 75 patients). Five years later, 12.0% (n = 9) of patients met the diagnosis of LLL. The break-down of the progression, regression and development of symptoms over the course of the study is depicted in Table 3. Of those who initially met the criteria of LLL by the GCLQ criteria (n = 11), 8 patients reported an improvement in symptoms while 3 patients had a persistent diagnosis of LLL. More importantly, 6 patients met the criteria of LLL on subsequent survey that had not previously met criteria. Therefore, over the course of the survey, 23% (n = 17) of patients were diagnosed with LLL at one time point.

**Table 2:** GCLQ Symptom Prevalence Reporting and diagnosis of LLL of patients who underwent surgical intervention in 2008–2010.

**GCLQ Symptom Cluster**	**Total Cohort: Initial Survey in 2011 (n = 205)**	**Follow up Cohort: Initial Survey in 2011 (n = 75)**	**Follow up Cohort: Follow up Survey in 2016 (n = 75)**	**McNemar Test**
**Physical Function**	98 (47.8%)	34 (45.3%)	44 (58.7%)	0.07
**Numbness**	137 (66.8%)	51 (68%)	45 (60%)	0.2
**General Swelling**	74 (36.1%)	27 (36%)	23 (30.7%)	0.37
**Infection**	24 (11.7%)	9 (12%)	9 (12%)	1.0
**Heaviness**	27 (13.2%)	9 (12%)	8 (10.7%)	0.76
**Aching**	111 (54.2%)	39 (52%)	39 (52%)	1.0
**Limb-Related Swelling**	14 (6.8%)	5 (6.7%)	5 (6.7%)	1.0
**Total GCLQ score**	2 (1, 4)	2 (1, 4)	2 (0, 4)	
**Diagnosis of LLL by GCLQ**	**23 (11.2%)**	**11 (14.7%)**	**9 (12%)**	**0.59**

**Table 3:** Timeline of Symptoms over 5 year follow up survey (n = 75).

**GCLQ Symptom Cluster**	**Physical Function**	**Numbness**	**General Swelling**	**Infection**	**Heaviness**	**Aching**	**Limb-Related Swelling**	**Diagnosis of LLL by GCLQ**
**Symptoms Present on Initial Survey (N = 75)**	**34 (45.3%)**	**51 (68%)**	**27 (36%)**	**9 (12%)**	**9 (12%)**	**39 (52%)**	**5 (6.7%)**	**11 (14.7%)**
Regression of Initial Symptoms	10 (29.4%)	14 (27.5%)	12 (44.4%)	7 (77.8%)	6 (66.6%)	14 (35.9%)	2 (40%)	8 (72.7%)
Persistence of Initial Symptoms	24 (70.6%)	37 (72.5%)	15 (55.6%)	2 (22.2%)	3 (33.3%)	25 (64.1%)	3 (60.0%)	3 (27.3%)
**No Symptoms Present on Initial Survey (N = 75)**	**41 (55.7%)**	**24 (32%)**	**48 (64%)**	**66 (88%)**	**66 (88%)**	**36 (48%)**	**70 (93.3%)**	**64 (85.3%)**
Developed New Symptoms	20 (48.8%)	8 (33.3%)	8 (16.7%)	7 (10.6%)	5 (7.6%)	14 (39%)	2 (2.9%)	6 (9.4%)
Never Had Symptoms	21 (51.2%)	16 (66.7%)	40 (83.3%)	59 (89.4%)	61 (92.4%)	22 (61%)	68 (97.1%)	58 (90.6%)
**Total Patients Affected Over Longitudinal Survey (N = 75)**	**54 (72%)**	**59 (79%)**	**35 (47%)**	**16 (21%)**	**14 (19%)**	**53 (71%)**	**7 (9%)**	**17 (23%)**

Most patients who completed the follow up survey (n = 75) experienced a persistence of symptoms in poor physical function (70.6%), numbness (72.5%), general swelling (55.6%), aching (64.1%), and limb-related swelling (60%). Infection (12%) and heaviness (12%) had a noted improvement after 5 years with regression in 77.8% and 66.6% of patients, respectively. Comparing initial versus follow up survey responses, new development of symptoms were reported among all of the GCLQ symptom clusters. There was noted development of symptoms among those patients previously unaffected within the physical function (20 of 41 patients), numbness (8 of 24 patients), and aching (14 of 36 patients)..

## 4 Discussion

We prospectively surveyed patients at 2 and 7 years after endometrial cancer treatment for symptoms of LLL. Using GCLQ criteria as a surrogate measure of LLL, the prevalence of LLL was 14.7% within 2.2 ± 1.2 years from diagnosis and treatment of endometrial cancer. Our study’s rate of LLL is similar to previously reported rates of 5% to 34.5%, however, when evaluated longitudinally, it was noted that many patients reported persistent symptoms in physical function (70.6%), numbness (72.5%), swelling (55.6%), and aching (64.1%). Further, the development of new symptoms years after initial diagnosis was noted in each of the subcategories with 14 of 36 (39%) previously unaffected patients developing aching and 20 of 41(48.8%) patients developing physical function concerns. (Abu-Rustum et al., 2006, Yost et al., 2014, Beesley et al., 2007, Brown et al., 2013, Ryan et al., 2003) The number of new diagnoses (N = 6) of LLL at follow up (7.2 ± 1.2 years from diagnosis) has not been previously reported. The new diagnoses at later times highlight the chronic nature of these symptoms and the long-term impacts of the diagnosis and treatment among endometrial cancer patients.

Although many patients did not meet the defined criteria for diagnosis of LLL, a significant portion of patients reported individual symptoms such as numbness and aching. Notably, 50% of patients reported poor physical function at each time point. The GCLQ questions are not specific enough to establish a definitive diagnosis or cause, but the high prevalence of symptoms in this population represents an opportunity to improve patient care.

LLL is often underdiagnosed clinically, partly stemming from a lack of awareness of the symptoms. Improving capture of LLL with earlier identification and referral to lymphedema programs could decrease the symptom burden for endometrial cancer survivors over an extended period of time. Studies have shown patient desire for improved education preoperatively on LLL. (Abu-Rustum et al., 2006, Yost et al., 2014, Beesley et al., 2007, Carter et al., 2021) Further, GOG 244 showed LLL symptoms negatively impact quality of life, daily activities, self-image, and can increase cancer distress. (Carter et al., 2021) Given the reported overall impact on quality of life measures and general decline in physical function, early awareness and symptom prevention could have significant impact for these patients. Of the patients who completed both surveys, 91% had lymphadenectomy with a median of 16 lymph nodes removed. Transitioning to sentinel lymph node biopsies may reduce LLL but this is yet to be confirmed. (Leitao et al., 2020 Jan) Further, proposed postoperative preventative measures including awareness, early referral to a lymphedema specialist, using caution during air travel with compressive measures, as well as possible improvements with exercise could be implemented. (Dessources et al., 2020, Brown et al., 2013, Brown et al., 2013) After diagnosis of lymphedema, complex rehabilitation programs may also assist in improving other quality of life measures for those diagnosed with LLL as well as have a positive impact on body image and mental health. (Jager et al., 2006, Do et al., 2017 Nov) Specifically, a small randomized controlled trial showed significant improvement in fatigue, pain, leg volume, and GCLQ scores for patients with LLL from gynecologic surgery who received a combination of manual lymphatic drainage, compression therapy, skin care, and remedial exercise. (Do et al., 2017 Nov).

Limitations of this study include possible volunteer bias with a survey-based approach. The survey time points did allow for capture of symptoms at two separate time points without recall bias retrospectively but increased the number of patients lost to follow up, with a response rate of 36% at second survey. Unfortunately, many of the symptoms associated with LLL, and those captured by the GCLQ, can be attributed in isolation to other medical comorbidities, treatments, or complications of aging can confound the diagnosis. (Dessources et al., 2020) For instance, the symptom of neuropathy may be due to lymphedema, effects of chemotherapy, or prior underlying condition such as diabetes. We also did not correlate the survey data with a global assessment of QOL. It is possible that these symptoms did not significantly affect a patient’s ability to perform activities of daily living, but this could be evaluated in future studies. This survey-based approach also did not capture the treatment and oncologic course for these patients, who may have needed further treatment, although most patients (80%) were Stage I. Thus, it is unclear how many of these patients received treatment for their lymphedema symptoms, or had progression of other comorbidities, such as obesity, that may have contributed to persistence of lymphedema symptoms.

Overall, this study contextualized the longitudinal impact and prevalence of LLL after surgery for endometrial cancer. Our study showed that the symptoms and burden of LLL persist and may even appear as late as 7 years after initial treatment for endometrial cancer. Given the known impacts on quality of life, this suggests a need for ongoing evaluation for symptoms with potential for intervention. Further research should target effective treatments, evaluation of the impact of comorbidities such as underlying neuropathy or obesity on persistent symptoms, and ongoing prevention of this debilitating condition and associated symptoms. While studies have shown the significant impact on daily activities, wardrobe, and financial burden associated with the diagnosis, (Ryan et al., 2003) the impact on the quality of life with some of these individual symptoms should be considered.

### CRediT authorship contribution statement

**Julia Ritchie:** Writing – original draft, Visualization. **Quetrell Heyward:** . **Nicholas Taylor:** Writing – review & editing. **Emily Ko:** Conceptualization, Methodology, Supervision. **Ashley F. Haggerty:** Conceptualization, Methodology, Supervision. **Ashley Graul:** Formal analysis, Data curation, Writing – review & editing, Supervision.

## Declaration of Competing Interest

The authors declare that they have no known competing financial interests or personal relationships that could have appeared to influence the work reported in this paper.
